# Carryover effects of baloxavir acid in human nasopharyngeal/pharyngeal swabs on infectious titer testing of influenza virus

**DOI:** 10.1111/irv.12721

**Published:** 2020-01-28

**Authors:** Keiko Baba, Ryoko Oka, Shinya Shano, Shinya Omoto, Takeshi Noshi, Takao Shishido, Akira Naito, Keiko Kawaguchi, Toru Ishibashi, Takeki Uehara

**Affiliations:** ^1^ Shionogi & Co., Ltd. Osaka Japan

**Keywords:** baloxavir acid, baloxavir marboxil, cap‐dependent endonuclease, drug carryover, influenza virus

## Abstract

Baloxavir marboxil (BXM) demonstrated a rapid and profound decline in infectious viral titer 1 day after BXM administration. Rapid reduction in virus titer is a characteristic of BXM. There may be a possibility that drug carryover effects have impacts on the observed antiviral effects due to the poor correlation that was observed between viral titer reduction and alleviation of influenza symptoms. Here, we report possible carryover effects of baloxavir acid (BXA), an active form of BXM, on infectious titer testing. Our findings indicate that there is little impact of BXA carryover on the infectious titer testing.

## 1 INTRODUCTION

Baloxavir marboxil (BXM), a prodrug of baloxavir acid (BXA), is in a new class of anti‐influenza drugs that is approved in Japan and the United States for the treatment of influenza A and B infections. Unlike neuraminidase inhibitors which suppress the release of new virus particles, BXA inhibits the cap‐dependent endonuclease activity which is essential for viral transcription in the early phase of viral replication.^1^, ^2^

In the phase 3 clinical trial involving otherwise healthy outpatients with acute uncomplicated influenza (CAPSTONE‐1, ClinicalTrials.gov number, NCT02954354), BXM treatment led to more rapid alleviation of influenza symptoms than placebo.^3^ In addition, BXM administration led to rapid and profound declines in infectious viral titers in nasopharyngeal/pharyngeal swabs at 1 day after a single oral dose (Day 2) compared with placebo and oseltamivir (OTV).^3^

Although the clinical trials comparing BXM to OTV showed similar reductions in influenza symptoms, treatment with BXM led to greater viral load reductions. This finding is due to the novel mechanism of action of BXM which results in rapid viral titer reduction, potentially leading to suppression of viral transmission. However, the carryover effects of BXA on the influenza virus have not yet been investigated. In this study, we assessed possible carryover effects of BXA, that is, the inhibition of virus growth in cells due to the higher inhibitor concentrations in the tested samples, on infectious titer testing.

## 2 MATERIALS AND METHODS

### 2.1 Study design

The CAPSTONE‐1 study was conducted in the United States and Japan as a double‐blind, placebo‐ and OTV‐controlled, randomized trial. The details of the trial have been reported previously.^3^

### 2.2 Quantification of BXA concentration in the nasal swab samples

Nasopharyngeal/pharyngeal swabs were placed into the universal transport medium (Puritan UniTranz‐RT^TM^) and immediately stored at 2 to 8°C.^4^ The swab samples were shipped to the central laboratory under cooled conditions, then subdivided, and stored at − 80°C. A total of 48 swab samples were randomly selected according to the following criteria: (a) collected from subjects at Day 2, (b) samples from the subjects whose plasma BXA concentration at Day 2 was more than 40.0 ng/mL, which was higher than 25th percentile for the total 589 of BXM‐treated subjects (34.3 ng/mL), and (c) stored at − 80°C within 96 hours after collection. To quantify the BXA concentration, 50 μL of the inoculated transport medium was mixed with acetonitrile and formic acid at the ratio of 1000:1 (v/v) for protein precipitation. The supernatants were subjected to liquid chromatography‐tandem mass spectrometry (LC‐MS/MS) analysis. The analytical method was validated for a quantification range of 0.0500 to 50.0 ng/mL.

### 2.3 Determination of BXA concentration that interferes with infectious titer testing

**Figure 1:**
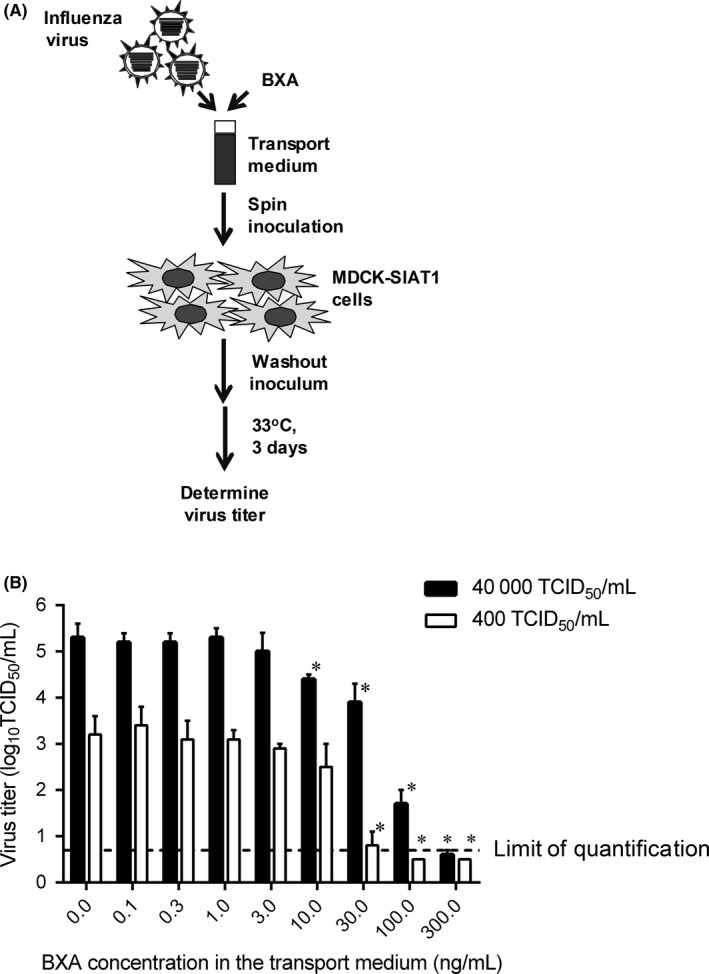
Viral titers of test virus samples in the Puritan transport medium including various concentrations of BXA determined in MDCK‐SIAT1 cells. (A) A schematic procedure of preparation of test virus samples and infectious virus titer testing. Test virus stocks of A/Victoria/361/2011 (H3N2) at 400 or 40 000 TCID_50_/mL (final concentration) and 0 to 300 ng/mL of BXA (final concentration) were diluted with the Puritan transport medium and mixed, followed by the determination of infectious viral titers (log_10_ TCID_50_/mL) in MDCK‐SIAT1 cells. (B) Viral titers of test virus samples. Data represent the mean and standard deviation of five experiments. The virus titers with lower limit of quantification (<0.7) were set as 0.5 log_10_ TCID_50_/mL for the calculation of statistics. * *P* < .05 to the baseline by Welch's *t* test

BXA was synthesized at Shionogi & Co., Ltd. (Osaka, Japan). A vaccine strain A/Victoria/361/2011 (H3N2)^5^ was diluted in the Puritan transport medium to reach the final viral titers at 400 or 40 000 50% tissue culture infectious dose (TCID_50_)/mL and mixed with BXA at the final concentration from 0 to 300 ng/mL; five‐independent portions were prepared for each sample (Figure 1A). The samples were stored at − 80°C according to the procedures used in the clinical trial. Viral titers were determined in MDCK‐SIAT1 cells^6^, ^7^ (European Collection of Cell Cultures) in the same way as in the CAPSTONE‐1 study.^3^ Briefly, the cells were inoculated with 10^0^‐ to 10^7^‐fold of diluted samples. The cells were absorbed by spin inoculation, followed by washing of the cells to remove unabsorbed viruses and BXA in the inoculum, and then, the cells were incubated at 33°C in a CO_2_ incubator for 3 days. The presence of virus‐induced cytopathic effects was evaluated, and the viral titers were determined by the Behrens‐Karber method (Figure 1B).^8^ The virus titer of less than the lower limit of quantification (LLOQ) was set as 0.5 log_10_ TCID_50_/mL. The comparisons of virus titers between the baseline without BXA and each BXA concentration were conducted by Welch's *t* test using SAS version 9.2 at *P* values of less than .05 significance level.

### 2.4 Comparison of declines between viral titers and RNA loads

Changes from the baseline in viral titers and RNA in the CAPSTONE‐1 study were quantified as described previously.^3^ Comparison of declines was conducted using data with viral titers and RNA higher than LLOQ (0.7 log_10_ TCID_50_/mL, or 2.18 log_10_ viral particle/mL). Correlations were analyzed by Pearson's product using SAS version 9.2 at *P* values of less than .05 significance level.

## 3 RESULTS

### 3.1 Quantification of BXA concentration in nasopharyngeal/pharyngeal swab samples from BXM‐treated subjects

The median plasma BXA concentration for the 48 selected subjects (72.1 ng/mL) was higher than that for the total 589 BXM‐treated subjects (51.5 ng/mL), indicating that this sample selection would not lead to underestimation of BXA concentration in the swab samples. LC‐MS/MS analysis determined that the median BXA concentration in the selected swab samples was 0.9655 ng/mL (5‐95 percentile, 0.08745‐2.4375 ng/mL) (Table 1).

**Table 1:** BXA concentration in plasma and nasopharyngeal/pharyngeal swab samples in the transport medium at Day 2 in the CAPSTONE‐1 study

	BXA concentration (ng/mL)		
	Plasma		Nasal swabs in transport medium
	All subjects	Selected subjects	Selected subjects
N	589	48	48
Range	0‐209.0	40.7‐209.0	<0.05‐3.78
Median	51.5	72.1	0.966
5th percentile	15.5	42.9	0.0875
95th percentile	119.5	164.2	2.44

Abbreviation: N, number of subjects.

### 3.2 Effects of BXA in the transport medium on influenza virus titer testing

When tested with the inoculum of 400 TCID_50_/mL, 30 ng/mL or higher BXA concentrations resulted in statistically significant reductions in influenza A(H3N2) viral titer compared with the sample without BXA, and 100 ng/mL or higher resulted in negative in viral titer (<LLOQ) (Figure 1B). With an inoculum of 40,000 TCID_50_/mL, 10 ng/mL or higher BXA concentration significantly affected the viral titer, and 300 ng/mL resulted in negative (Figure 1B).

### 3.3 Comparison of declines between viral titers and RNA loads

**Figure 2:**
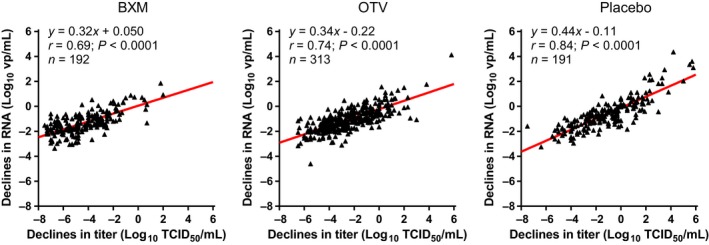
Correlation of declines between infectious viral titers and RNA loads at Day 2 in the CAPSTONE‐1 study. Horizontal axis indicates declines from baseline (at Day 1) in viral titers, and vertical axis indicates declines from baseline in viral RNA loads at Day 2 from BXM‐, OTV‐, and placebo‐treated patients in the CAPSTONE‐1 study. Viral titers and RNA loads higher than lower quantification limit (0.7 log_10_ TCID_50_/mL, or 2.18 log_10_ vp/mL) were used for analysis. Pearson's correlation coefficient (*r*) in BXM‐, OTV‐, and placebo‐treated groups were 0.69 (*P* < .0001), 0.74 (*P* < .0001), and 0.84 (*P* < .0001), respectively. Regression line (redline) and the number of samples (n) are also shown

We next compared declines in infectious viral titers with RNA at Day 2 on the total BXM‐treated cohort (n = 192). Overall, viral RNA decreased in proportion to viral titer reductions in all groups, and Pearson's correlation analysis showed a significant positive correlation of declines in viral titers versus RNA in BXM groups (Pearson *r* = .69; *P* < .0001) (Figure 2).

## 4 DISCUSSION

In this study, we found that the median BXA concentration in the swab samples was 0.9655 ng/mL at Day 2. Considering that approximately 0.1 mL was collected on a swab and diluted 40‐fold in 4.0 mL of the transport medium, it was estimated that roughly 40 ng/mL of BXA was delivered in the swabs, which is comparable to a plasma level of 71.5 ng/mL, in the selected samples. In addition, there is little possibility that BXA was degraded during the storage. While the swab samples used in this study were stored for at most 287 days, we have revealed that BXA was stable at − 20°C/−80°C for up to 296 days in the Puritan transport medium. Although the exact reason is unclear for the absence of a significant correlation in BXA concentration between swab and plasma (Pearson *r *= .19; Figure S1), it is speculated that the difference in the collected volume of nasal swabs and sampling methods might account for the result. Nevertheless, the data shown here demonstrated that BXA was delivered to the throat or nasal cavity, that is virus replication site, after oral BXM administration.

We revealed that significant interference could have occurred when 10.0 ng/mL or higher BXA concentration was present in the swab samples. Although the median BXA concentration in the swab samples (0.9655 ng/mL) was comparable to the EC_50_ of BXA against baseline viruses (0.31 to 0.69 ng/mL) in the CAPSTONE‐1 study,^9^ the median BXA concentration did not significantly affect the virus titer. This may be due to the washout step being utilized to remove uninoculated viruses and BXA according to the protocol used for all subjects’ samples in the clinical trials.^3^

In the previous report, a significant decline was observed in both viral titers and viral RNA in the BXM‐treated group compared with the placebo and OTV groups.^3^ Comparative analysis confirmed a significant correlation between viral titers and RNA in total BXM group at Day 2 (*r* = .69). In addition, we confirmed the absence of a significant correlation between BXA concentration in the swab samples and the viral titer reduction in the selected subjects (*r* = .032; Figure S2). These data indicate that BXA in the swab samples does not interfere with the infectious titer testing. In addition, these findings provide the rationale to continue to evaluate BXM’s antiviral effects in samples collected from other clinical trials, such as from high‐risk influenza patients, and in a trial investigating the suppression of viral transmission. 

A limitation of this study was that we tested with only A(H3N2) strain and did not test A(H1N1)pdm and type B viruses. This is because 86.2% of patients in BXM group were infected with influenza A(H3N2) in the CAPSTONE‐1 study.^3^ Considering that viral strains and BXA concentrations detected in nasal secretions were different for each subject, a possible carryover effect cannot be excluded in some subjects. However, BXA displays comparable inhibitory potency against A(H3N2) and A(H1N1) viruses, while type B viruses are less susceptible to BXA.^1^, ^2^, ^10^, ^11^, ^12^, ^13^ Therefore, it could be assumed that the data shown here are not likely to underestimate the carryover effects of BXA.

In conclusion, our findings indicate that there is very little impact of BXA carryover on the infectious titer tested in nasopharyngeal/pharyngeal swab samples in the CAPSTONE‐1 study. This study supports the clinical evidence on rapid and profound decline of viral titer in BXM‐treated patients.

## AUTHOR CONTRIBUTIONS

KB, RO, SO, TN, TS, AN, and TU contributed to project design, data analysis, and interpretation. KB and SS involved in determination of BXA concentration that interferes with infectious titer testing. RO involved in quantification of BXA concentration in the swabs. KB, TS, KK, TI, and TU performed analyses on clinical data. KB, SO, and TU wrote manuscript. All authors read and approved the manuscript.
