# Supplementary material for: Carryover effects of baloxavir acid in human nasopharyngeal/pharyngeal swabs on infectious titer testing of influenza virus
Source: Influenza Other Respir Viruses. 2020 Jan 28;14(3):353–7. doi: 10.1111/irv.12721 (PMC7182602; doi:10.1111/irv.12721)
Supplement: Supplementary file 1 [file IRV-14-353-s001.docx]

**APPENDICES**

**SUPPLEMENTARY FIGURE 1.** Correlation of BXA concentrations between plasma and swab samples. A total of 48 subjects were selected and the BXA concentrations of their swab samples were measured. BXA concentration of selected samples in plasma (vertical axis) and in swab samples (horizontal axis) are plotted in this figure. BXA concentration in swab samples with lower limit of quantification (< 0.05) were set as 0.05 ng/mL in this figure. Pearson correlation coefficient (r), regression line (redline), and the number of samples (n) are also shown.

**

**

**SUPPLEMENTARY FIGURE 2.** Correlation between BXA concentration in swab samples and declines from baseline (at Day 1) in infectious virus titers at Day 2. A total of 48 subjects were selected and the BXA concentrations in their swab samples were measured. Horizontal axis indicates BXA concentration in swab samples and vertical axis indicates declines in infectious virus titers at Day 2 from selected subjects in the CAPSTONE-1 study. Among 48 tested subjects, data from 22 subjects with BXA concentration in swab samples and viral titers higher than lower quantification limit (0.05 ng/mL, or 0.7 log_10_ TCID_50_/mL) were used for the analysis. Pearson correlation coefficient (r), regression line (redline), and the number of samples (n) are also shown.
